# Non-Genetic Factors and Risk of Cervical Cancer: An Umbrella Review of Systematic Reviews and Meta-Analyses of Observational Studies

**DOI:** 10.3389/ijph.2023.1605198

**Published:** 2023-03-31

**Authors:** Xin-Yu Li, Gang Li, Ting-Ting Gong, Jia-Le Lv, Chang Gao, Fang-Hua Liu, Yu-Hong Zhao, Qi-Jun Wu

**Affiliations:** ^1^ Department of Clinical Epidemiology, Shengjing Hospital of China Medical University, Shenyang, China; ^2^ Clinical Research Center, Shengjing Hospital of China Medical University, Shenyang, China; ^3^ Department of Ultrasound, Shengjing Hospital of China Medical University, Shenyang, China; ^4^ Department of Obstetrics and Gynecology, Shengjing Hospital of China Medical University, Shenyang, China

**Keywords:** risk factors, cervical cancer, evidence, meta-analyses, umbrella review

## Abstract

**Objectives:** The association between non-genetic risk factors and cervical cancer (CC) remains controversial and unclear. This umbrella review was conducted to evaluate and synthesize previously published systematic reviews and meta-analyses related to non-genetic factors and CC risk.

**Methods:** We searched PubMed, Web of Science, and EMBASE to identify studies investigating the association between extragenetic factors and CC risk. For each article, we calculated the summary effect size and the 95% confidence interval. Specific criteria were used to classify the association into four levels: strong, highly suggestive, suggestive, or weak.

**Results:** A total of 18 meta-analyses of different risk factors for CC were examined; these studies covered risk factors related to diet, lifestyle, reproduction, disease, viral infection, microorganisms, and parasites. Oral contraceptive use and *Chlamydia trachomatis* infection were shown to increase CC risk, and this was supported by strong evidence. Additionally, there were four risk factors supported by highly suggestive evidence and six risk factors supported by suggestive evidence.

**Conclusion:** In conclusion, there is a strong association between oral contraceptive use, *Chlamydia trachomatis* infection, and increased CC risk.

## Introduction

Cervical cancer (CC) is one of the most severe malignancies affecting women. In 2020, CC was the fourth most frequently diagnosed cancer and the fourth leading cause of cancer death in women, with an estimated 604,000 new cases and 342,000 deaths worldwide (1). Resulting from lack of resources and the absence of effective interventions, CC is very common in low- and middle-income countries (2). The occurrence and development of this disease is a continuous process, and intervention is focused on primary and secondary prevention (3). In high-income countries, screening and treatment of pre-cancerous lesions are common preventive approaches. However, CC remains the most common cause of cancer mortality amongst women (4), which suggests that it is necessary to identify risk factors to improve CC prevention. Previous epidemiologic studies have demonstrated persistent infection with human papillomavirus (HPV) is reported the primary cause of CC (5).

Recent evidence has identified some non-genetic risk factors of CC, such as oral contraceptive use, smoking, household air pollution, and trichomonas vaginalis infection (6–9). Oral contraceptive use can increase CC risk, especially for longer duration of oral contraceptive use (10). A meta-analysis around 14 studies showed that passive smoking is a risk factor for CC (7). Eight human papillomavirus genotypes (HPV16, 18, 31, 33, 35, 45, 52 and 58) bring about a higher CC risk than others in a study in Japan (11). Zhang et al indicated that serum copper levels in patients with CC higher than in controls, which means serum copper may aggrandize CC risk (12). However, systematic reviews and meta-analyses reported different findings related to extragenetic factors in CC, including lifestyle, virus, reproductive factors, diseases, and nutrition and nutrient levels (7, 13, 14). These conflicting results are attributed to incomplete evaluations and influence from other biases. Umbrella reviews can systematically elucidate the strength of existing evidence and evaluate the risk of bias in published systematic reviews and meta-analyses on CC risk factors (15). To date, no conclusive umbrella reviews have been completed to evaluate the association between extragenetic factors and CC risk in humans.

In this context, we conducted an umbrella review of current systematic reviews as well as meta-analyses of observational studies to systematically assess the strength and validity of the association between extragenetic factors and CC risk (16). We performed an umbrella review to comprehensively summarize all available evidence regarding the association between extragenetic factors and CC risk in humans using a standardized approach. We also evaluated hints of bias in these associations. Ultimately, we confirmed robust epidemiologic evidence that has been previously reported in meta-analyses.

## Methods

### Literature Search and Eligibility Criteria

We completed an umbrella review, which is a systematic collection and assessment of multiple systematic reviews and meta-analyses of a specific research topic (17). We searched PubMed, Web of Science, and Embase for related systematic reviews and meta-analyses published from inception to October 12, 2020. The preset search strategy is shown in Supplementary Table S1. This study was registered at PROSPERO (No. 42021236238).

### Inclusion and Exclusion Criteria

Each systematic review and meta-analysis were reviewed independently by two authors (X-YL and CG) to identify studies that met the inclusion criteria. Differences were resolved by a third author (Q-JW). Inclusion criteria were as follows: 1) systematic reviews and meta-analyses of observational studies that assessed the association between extragenetic factors and CC risk in humans; 2) studies that provided effect sizes [odds ratio (OR), risk ratio (RR), hazard ratio (HR)] of CC for various extragenetic factors; and 3) articles that were published in English. If multiple systematic reviews and meta-analyses discussing the same exposures and outcomes were found, we included the one with the largest number of original studies (18). We included information about all extragenetic factors in CC that we were of interest in each study, including subgroup analysis and dose-response analysis.

Articles were excluded if they met the following criteria: 1) study exposure and outcome were not of interest; 2) studies using animals; or 3) articles that did not report necessary study-specific data [e.g., risk estimates, 95% confidence intervals (CIs)]. Systematic reviews and meta-analyses that only reported pre-cancerous lesions (cervical intraepithelial neoplasia, CIN) or that assessed pre-cancerous lesions in combination with CC were also excluded.

### Data Extraction

Two authors (X-YL and CG) extracted data independently. When discrepancies arose, the decision was made by a third author (Q-JW). We employed a data-collection form to acquire data from eligible studies. The data-collection form included both the information of the original study and the information of the meta-analysis. For meta-analyses: first author, publication year, number of included studies, exposure, outcome, case number, total population, and comparison were recorded. For original studies: study design, case number, total population, most adjusted risk estimates (RR, OR, HR), and corresponding 95% CIs were recorded.

### Statistical Analysis

#### Estimation of Summary Effect and Heterogeneity

We calculated the summary effect size and 95% CI for each exposure and outcome using both fixed-effects models and DerSimonian-Laird random-effects models (19, 20). Heterogeneity was evaluated using the I^2^ statistic, with I^2^ ≥ 50% or I^2^ ≥ 75% representing large heterogeneity and very large heterogeneity, respectively. Due to the uncertainty of heterogeneity between studies, we further calculated the 95% CI of I^2^ (21). After completing the above calculations, we explored the 95% prediction interval to estimate the expected effect size range in the new primary studies, which further accounted for between-study heterogeneity and assessed uncertainty for the effect in the random effects model (22).

#### Assessment of Small-Study Effects

Small-study effects indicate whether smaller studies tend to give substantially larger estimates of effect size compared with larger studies, and this can be estimated by Egger’s regression asymmetry test (23). This can reflect publication bias, differences between small and large studies on account of genuine chance, heterogeneity, among other reasons. The criteria of small-study effects were: 1) Egger’s test *p* < 0.1; and 2) effect size in the largest study was smaller than the summary effect size (24). We calculated standard error to evaluate the largest study of each meta-analysis and determine whether it met the criteria of small-study effects.

#### Evaluation of Excess Significance

In consideration of the relative excess of formally significant findings in published literature, we employed the excess of statistical significance test (25). We assessed excess significance by inquiring whether the number of observed studies with statistically significant results (O) was larger than the expected number (E). The expected number of studies with significant results (E) was calculated by the sum of the statistical power estimates for each component study in each of the meta-analyses. The power of each component study was estimated using the fixed effects summary, the random effects summary, or the effect size of the largest study (smallest standard error) as the plausible effect size (25, 26). The power of each study was calculated with an algorithm using a non-central *t* distribution (27). Excess significance was based on both O > E and *p* < 0.1 (28). Statistical analysis was performed in STATA 15.0.

### Evaluation of Evidence in the Included Meta-Analyses

We used specific criteria to grade the association between extragenetic factors and CC risk. Our criteria for evaluation of evidence in concordance with the strategies used in previously published umbrella reviews (29–31). There were four levels of evidence: strong, highly suggestive, suggestive, and weak (Supplementary Table S3). The criteria for strong evidence included random effects *p* < 10^−6^, number of cases >1,000, I^2^ < 50%, *p* < 0.05 of the largest study in the meta-analysis, 95% prediction interval excluding the null value, absence of small-study effects (*p* > 0.1 for Egger’s test), and no excess significance bias (*p* > 0.1). The criteria for highly suggestive evidence included random effects *p* < 10^−6^, number of cases >1,000, and *p* < 0.05 of the largest study in the meta-analysis. The criteria for suggestive evidence included random effects *p* < 10^−3^ and number of cases >1,000. The sole criterion for weak evidence was random effects *p* < 0.05. In cases with *p* > 0.05, there was no association.

### Evaluation of the Quality of Included Meta-Analyses

Two authors independently evaluated the quality of each included systematic review and meta-analysis. The evaluation was based on the Assessment of Multiple Systematic Reviews (AMSTAR) tool, which provides 11 items to measure the methodological quality of meta-analyses (32); the higher the total score, the higher the quality of the report. A review scoring above 8 is graded as high quality, 4–7 is moderate quality, and below 4 is low quality.

## Results

### Literature Search

**FIGURE 1:**
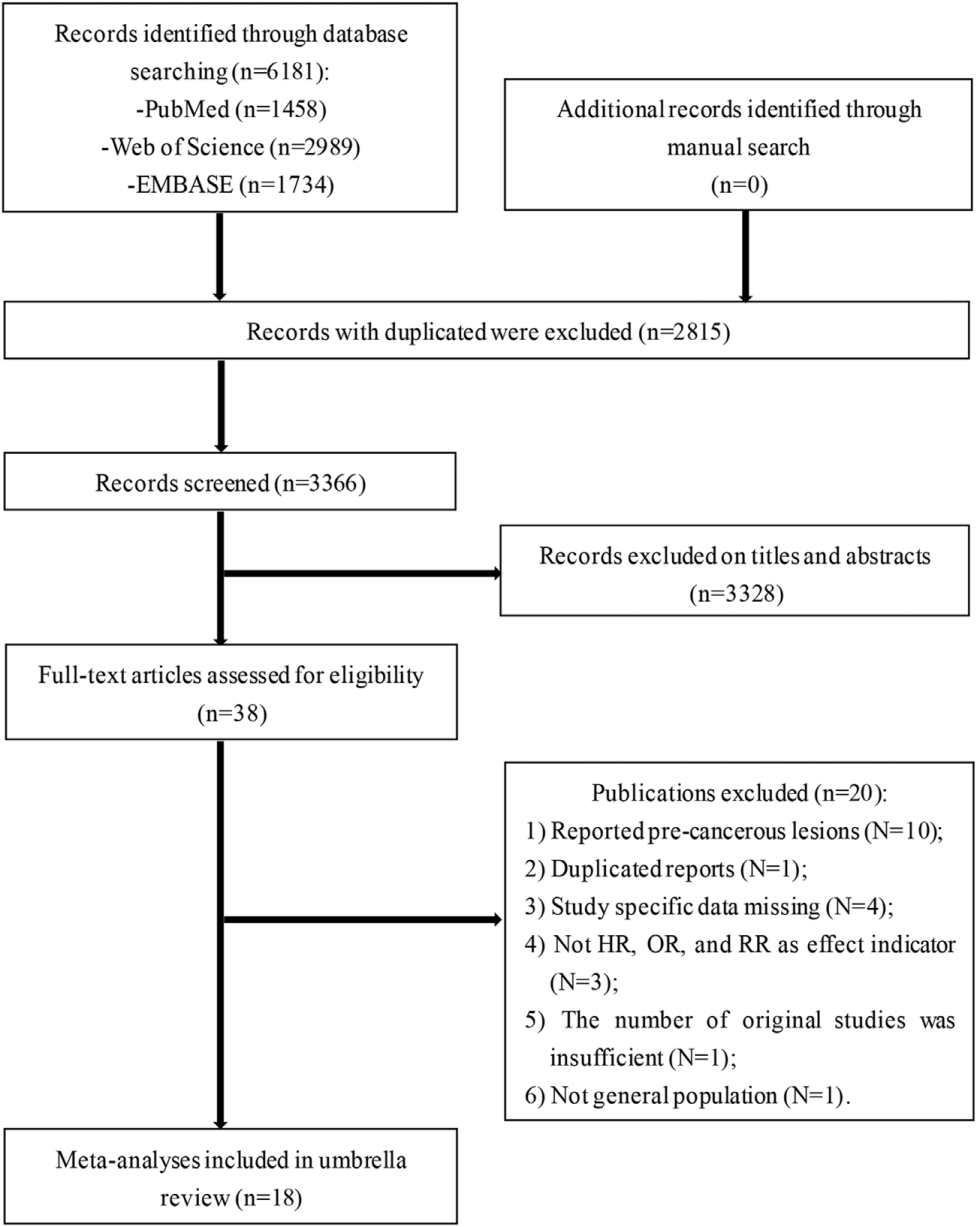
Flowchart of selection of studies for inclusion in umbrella review on non-genetic factors and risk of cervical cancer (Liaoning, China. 2020).

The literature search strategy retrieved 6,181 publications from three electronic databases (Figure 1). After removing duplicates, preliminary screening was conducted using titles and abstracts and a total of 38 records were deemed eligible. Twenty references were excluded for various reasons upon reviewing the full-text articles (Figure 1). Ultimately, 18 articles were included for the final analysis.

### Description of Eligible Meta-Analyses

A total of 39 different associations between non-genetic risk factors and CC risk were examined in the 18 meta-analyses (Table 1) (10, 33–49). These risk factors were grouped into five broad categories: lifestyle factors (smoking, overweight, and obesity); reproductive factors (*in vitro* fertilization, intrauterine devices, and oral contraceptives); disease factors (endometriosis, gestational diabetes mellitus); dietary intake factors (vitamin A, vitamin E, and selenium); and virus, microorganism, and parasite factors (HPV, herpes simplex type 2, *C. trachomatis*, Epstein-Barr virus, and cervicovaginal lactobacilli). All articles were published between 2001 and 2020. Of the 18 studies, the median number of original studies in each systematic review or meta-analysis was four (range: 3–16). The study design of the synthesized studies included case-control, cohort, nested case-control, cross-sectional, and friend/family studies. The number of cases and participants ranged from 23 to 4,945 and from 111 to 5,371,295, respectively. Outcome indicators included various types of CC, such as adenocarcinomas of the cervix, squamous cell carcinoma, invasive CC, cervical carcinoma *in situ*, and early stage CC.

**TABLE 1:** Characteristics of the eligible meta-analyses of multiple risk factors for cervical cancer (Liaoning, China. 2021).

Risk factor	Individual study	No. of studies	Effect metric	Outcome	Level of comparison	Study design
**Life style**						
Past smokers	(33)	3	OR	Adenocarcinoma	Past vs. never smokers	Case control
Current smokers		3	OR		Current vs. never smokers	
Past smokers		3	OR	Squamous cell carcinoma	Past vs. never smokers	
Current smokers		3	OR		Current vs. never smokers	
Past smokers	(34)	10	RR	Adenocarcinoma	Past vs. never smokers	Case control
Current smokers		10	RR		Current vs. never smokers	
Overweight	(35)	9	OR	Cervical cancer	Overweight vs. reference	Case control, cross sectional, and cohort
Obesity		9	OR		Obesity vs. reference	
Smoking	(36)	5	RR	Cervical cancer	Smokers vs. never smokers	Case control and cohort
**Virus, microorganism, and parasite**						
HPV and HPV16	(37)	3	RR	Invasive cervical cancer/Cervical carcinoma in situ	Exposed vs. unexposed	Case control
		4	RR			
Herpes simplex type 2	(38)	16	OR	Cervical cancer	Exposed vs. unexposed	Nested case control and case control
HPV16 A4/Asian variants	(39)	7	OR	Invasive cervical cancer	A4 vs. A1-3 variants	Case control
*Chlamydia trachomatis* infection	(40)	3	OR	Cervical cancer	Exposed vs. unexposed	Case control, nested case control, cross sectional, and cohort
		16	OR			
Coinfection of HPV and *Chlamydia trachomatis*		6	OR			
*Chlamydia trachomatis* infection		10	OR	Squamous carcinoma		
		4	OR	Adenocarcinoma		
*Chlamydia trachomatis* infection (serum)		15	OR	Cervical cancer		
Epstein-Barr virus	(41)	9	OR	Cervical cancer	Exposed vs. unexposed	Case control
Cervicovaginal lactobacilli	(42)	3	OR	Cervical cancer	Cervicovaginal lactobacilli vs. non-lactobacilli-predominant CST IV	Cross sectional
**Reproductive factors**						
*In vitro* fertilization	(43)	4	RR	Cervical cancer	Exposed vs. unexposed	Cohort
Intrauterine device use	(44)	10	OR	Cervical cancer	Intrauterine Device Use vs. no use	Friend/Family and cohort
Oral contraceptives use	(10)	4	OR	Cervical carcinoma in situ	Exposed vs. unexposed	Case control and cohort
		4	OR	Cervical adenocarcinoma		
		5	OR	Squamous cell carcinoma		
Oral contraceptives >10 years		3	OR	Cervical cancer		
**Diseases**						
Endometriosis	(45)	3	RR	Cervical cancer	Exposed vs. unexposed	Cohort
Gestational diabetes mellitus	(46)	3	RR	Cervical cancer	Exposed vs. unexposed	Cohort
**Nutrients and their levels**						
Total vitamin A intake	(47)	11	OR	Cervical cancer	Highest vs. lowest	Case control
Blood vitamin A levels (retinol)		3	OR			
Blood vitamin A levels (carotene)		4	OR			
Retinol intake		8	OR			
Carotene intake		7	OR			
Carotenoid intake		3	OR			
Retinol intake		3	OR	Early stage cervical cancer		
Carotene intake		3	OR			
Serum selenium levels	(48)	5	OR	Cervical cancer	Highest vs. lowest	Case control
Vitamin E	(49)	8	OR	Cervical cancer	Highest vs. lowest	Case control

Abbreviation: CI, confidence interval; CST, community state types; HPV, human papillomavirus; HR, Hazard ratio; OR, Odds ratio; RR, Relative risk.

All statistical tests were two-sided.

### Summary Effect Size

In this study, we used the random-effects model and the fixed-effects model to re-analyze 39 associations in the 18 meta-analyses. When *p* < 0.05 was used as the threshold for statistical significance, the summary fixed-effects and random-effects estimates were significant in 31 (79%) and 29 (74%) of the meta-analyses, respectively. Of these associations, 18 reported increased risks of CC and 11 showed decreased risks of CC under the random effects model. A total of 18 (46%) associations generated significant summary results (*p* < 0.001) using the random-effects model, including smoking, HPV and HPV16, herpes simplex type 2, *C. trachomatis*, Epstein-Barr virus, intrauterine devices, oral contraceptives, endometriosis, vitamin A, vitamin E, and selenium intake. A total of 25 (64%) associations showed statistically significant effects (*p* < 0.001) using the fixed-effects model. At a more stringent threshold of significance (*p* < 1 × 10^−6^), the summary random-effects estimates were significant for nine (23%) associations and the summary fixed-effects estimates were significant for 18 (46%) meta-analyses. Of the nine associations, eight meta-analyses (smoking, HPV and HPV16, *C. trachomatis*, and oral contraceptives) showed increased CC risk, and only vitamin A showed decreased CC risk. The summary random effect estimates are presented in Table 2 and range from 0.16 to 16.55. The largest study had the smallest SE for each association (Table 2). Twenty-four (62%) risk factors of the 39 associations showed statistically significant effects at *p* < 0.05.

**TABLE 2:** Quantitative synthesis of the eligible meta-analyses of multiple risk factors for cervical cancer (Liaoning, China. 2021).

Risk factor (reference)	No. of cases/participants	Summary relative risk (95% CI)			Random *p* value^b^	Fixed *P* value^c^
		Random effects	Fixed effects	Largest study^a^		
**Life style**						
Past smokers	316/1363	0.87 (0.63–1.22)	0.87 (0.63–1.22)	0.75 (0.46–1.20)	0.432	0.432
Current smokers	352/1565	0.84 (0.53–1.33)	0.82 (0.60–1.12)	0.82 (0.56–1.21)	0.461	0.213
Past smokers	1658/2705	1.07 (0.61–1.87)	0.94 (0.70–1.27)	0.70 (0.47–1.03)	0.825	0.705
Current smokers	1978/3191	1.57 (1.10–2.24)	1.47 (1.15–1.88)	1.26 (0.93–1.71)	0.014	0.002
Past smokers	1417/15292	0.92 (0.75–1.14)	0.92 (0.75–1.14)	0.75 (0.53–1.07)	0.447	0.447
Current smokers	1417/15292	0.90 (0.72–1.12)	0.90 (0.75–1.08)	0.81 (0.58–1.13)	0.336	0.263
Overweight	2557/5371295	1.10 (0.97–1.25)	1.10 (1.04–1.17)	1.10 (1.03–1.17)	0.146	0.002
Obesity	2557/5371295	1.45 (1.15–1.83)	1.28 (1.15–1.42)	1.21 (1.06–1.37)	0.001	3.634 × 10^−6^
Smoking	1316/231002	2.10 (1.60–2.77)	1.92 (1.68–2.20)	1.57 (1.30–1.89)	1.274 × 10^−7^	6.226 × 10^−22^
**Virus, microorganism, and parasite**						
HPV and HPV16	217/466	16.55 (8.22–33.33)	16.55 (8.22–33.33)	17.00 (6.80–44.00)	3.861 × 10^−15^	3.861 × 10^−15^
	260/763	3.68 (2.01–6.75)	3.26 (2.15–4.94)	3.20 (1.70–6.20)	2.389 × 10^−5^	2.383 × 10^−8^
Herpes simplex type 2	3337/10047	1.21 (1.04–1.41)	1.21 (1.04–1.41)	1.10 (0.80–1.40)	0.015	0.015
HPV16 A4/Asian variants	449/565	2.81 (1.44–5.51)	2.67 (1.89–3.76)	1.72 (1.04–2.85)	0.003	2.029 × 10^−8^
*Chlamydia trachomatis* infection	832/4305	2.21 (1.62–3.03)	2.22 (1.88–2.61)	2.21 (1.84–2.65)	6.427 × 10^−7^	2.625 × 10^−21^
	3459/7494	2.19 (1.74–2.74)	2.19 (1.95–2.45)	2.44 (2.06–2.89)	1.028 × 10^−11^	2.047 × 10^−41^
Coinfection of HPV and *Chlamydia trachomatis*	1086/4608	4.37 (2.75–6.96)	3.75 (2.92–4.82)	3.23 (2.39–4.35)	4.593 × 10^−10^	6.104 × 10^−25^
*Chlamydia trachomatis* infection	3198/8618	2.09 (1.79–2.44)	2.21 (1.99–2.45)	2.55 (2.15–3.03)	6.866 × 10^−21^	3.973 × 10^−51^
	329/2162	1.60 (1.19–2.14)	1.60 (1.19–2.14)	1.46 (0.96–2.23)	0.002	0.002
*Chlamydia trachomatis* infection (serum)	3528/10421	2.15 (1.83–2.53)	2.19 (2.00–2.41)	2.44 (2.06–2.89)	8.143 × 10^−21^	2.736 × 10^−59^
Epstein-Barr virus	398/1062	4.00 (1.89–8.50)	2.94 (2.07–4.16)	0.20 (0.08–0.48)	3.050 × 10^−4^	1.311 × 10^−9^
Cervicovaginal lactobacilli	23/111	0.16 (0.05–0.51)	0.16 (0.05–0.51)	0.18 (0.04–0.78)	0.002	0.002
**Reproductive factors**						
In vitro fertilization	174/40524	1.07 (0.45–2.55)	0.64 (0.55–0.74)	0.61 (0.52–0.71)	0.871	5.010 × 10^−9^
Intrauterine device use	4945/12482	0.64 (0.53–0.77)	0.70 (0.63–0.79)	0.89 (0.73–1.08)	1.906 × 10^−6^	5.409 × 10^−9^
Oral contraceptives use	2654/33389	1.70 (1.18–2.44)	1.42 (1.25–1.62)	1.34 (1.16–1.55)	0.004	1.311 × 10^−7^
	651/5660	1.77 (1.40–2.24)	1.77 (1.40–2.24)	1.60 (1.20–2.13)	1.625 × 10^−6^	1.625 × 10^−6^
	3331/22205	1.29 (1.18–1.42)	1.29 (1.18–1.42)	1.31 (1.19–1.44)	2.436 × 10^−8^	2.436 × 10^−8^
Oral contraceptives >10 years	154/308226	2.24 (1.45–3.48)	2.24 (1.45–3.48)	2.93 (1.44–5.96)	3.125 × 10^−4^	3.125 × 10^−4^
**Diseases**						
Endometriosis	203/192501	0.67 (0.54–0.84)	0.67 (0.54–0.84)	0.71 (0.53–0.94)	4.398 × 10^−4^	4.398 × 10^−4^
Gestational diabetes mellitus	1308/1163875	1.02 (0.81–1.29)	1.02 (0.81–1.29)	0.90 (0.65–1.26)	0.843	0.843
**Nutrients and their levels**						
Total vitamin A intake	3418/10478	0.59 (0.49–0.72)	0.62 (0.57–0.68)	0.95 (0.74–1.22)	1.750 × 10^−7^	8.062 × 10^−27^
Blood vitamin A levels (retinol)	236/707	1.14 (0.83–1.57)	1.14 (0.83–1.57)	1.14 (0.78–1.66)	0.422	0.422
Blood vitamin A levels (carotene)	474/1504	0.48 (0.30–0.76)	0.57 (0.45–0.71)	0.79 (0.54–1.14)	0.002	1.495 × 10^−6^
Retinol intake	1078/3227	0.80 (0.64–1.00)	0.85 (0.73–0.99)	0.95 (0.74–1.22)	0.048	0.042
Carotene intake	1244/4053	0.51 (0.35–0.74)	0.48 (0.42–0.55)	0.62 (0.47–0.80)	3.150 × 10^−4^	1.446 × 10^−23^
Carotenoid intake	603/1754	0.60 (0.43–0.84)	0.60 (0.49–0.74)	0.60 (0.46–0.78)	0.003	2.029 × 10^−6^
Retinol intake	371/761	0.83 (0.62–1.10)	0.83 (0.62–1.10)	0.82 (0.56–1.19)	0.193	0.193
Carotene intake	434/895	0.37 (0.18–0.73)	0.29 (0.22–0.38)	0.21 (0.15–0.30)	0.004	1.657 × 10^−18^
Serum Selenium levels	353/1206	0.55 (0.42–0.73)	0.55 (0.42–0.73)	0.58 (0.37–0.91)	2.215 × 10^−5^	2.215 × 10^−5^
Vitamin E	1321/4177	0.53 (0.39–0.73)	0.55 (0.48–0.63)	0.48 (0.38–0.61)	7.325 × 10^−5^	9.279 × 10^−18^

Abbreviation: CI, confidence interval; HPV, human papillomavirus.

^a^ Relative risk and 95% confidence interval of largest study (smallest SE) in each meta-analysis.

^b^ *p* value of summary random effects estimate.

^c^ *p* value of summary fixed effects estimate.

All statistical tests two sided.

### Heterogeneity and 95% Prediction Intervals

The heterogeneity of the 39 associations was evaluated using the I^2^ statistic. Twenty-six (67%) showed low heterogeneity (I^2^ < 50%), eight (20%) meta-analyses had large heterogeneity (I^2^ = 50–75%), and five (13%) had very large heterogeneity (I^2^ > 75%). We calculated the 95% prediction intervals, and the null value was excluded in eight (20%) meta-analyses; this included herpes simplex type 2, *C. trachomatis* infection, oral contraceptive use, and selenium intake. Heterogeneity and 95% prediction intervals are shown in Table 3.

**TABLE 3:** Level of evidence for the association of risk factors for cervical cancer (Liaoning, China. 2021).

Risk factor	Features used for classification of level of evidence								Evidence class^e^
	Significance threshold reached^a^	I^2^ (95% CI)	95% prediction interval	Egge’s *p* value	Excess significance^b^		Largest study significant	Small-study effect/excess significant bias	
					O/E^c^	*p* value^d^			
**Life style**									
Past smokers	>0.05	0 (0–90)	(0.10–7.68)	0.733	0/0.3406	0.5354	No	No/No	NS
Current smokers	>0.05	37 (0–80)	(0.01–68.02)	0.657	0/0.5316	0.4215	No	No/No	NS
Past smokers	>0.05	68.4 (0–91)	(0.00–615.58)	0.251	0/1.1615	0.1686	No	No/No	NS
Current smokers	<0.05 but >0.001	42.7 (0–83)	(0.05–53.22)	0.343	1/0.7286	0.7148	No	No/No	Ⅳ
Past smokers	>0.05	0 (0–62)	(0.72–1.18)	0.479	0/1.8199	0.1358	No	No/No	NS
Current smokers	>0.05	20 (0–61)	(0.58–1.39)	0.599	0/1.9952	0.1144	No	No/No	NS
Overweight	>0.05	19.9 (0–61)	(0.85–1.42)	0.939	2/1.6457	0.7600	Yes	No/No	NS
Obesity	<0.05 but >0.001	57.2 (10–80)	(0.78–2.72)	0.170	3/2.2797	0.5809	Yes	No/No	Ⅳ
Smoking	<10^−6^	64 (5–86)	(0.89–4.96)	0.434	4/2.1087	0.0868	Yes	No/Yes	Ⅱ
**Virus, microorganism, and parasite**									
HPV and HPV16	<10^−6^	0 (0–90)	(0.18–1547.16)	0.121	3/0.1514	0.0000	Yes	No/Yes	Ⅳ
	<0.001 but >10^−6^	43.8 (0–81)	(0.42–32.51)	0.088	4/0.8277	0.0001	Yes	Yes/Yes	Ⅳ
Herpes simplex type 2	<0.05 but >0.001	0 (0–48)	(1.03–1.43)	0.428	1/2.9113	0.2155	No	No/No	Ⅳ
HPV16 A4/Asian variants	<0.05 but >0.001	62.1 (18–32)	(0.42–18.99)	0.896	3/2.2465	0.5418	Yes	No/No	Ⅳ
*Chlamydia trachomatis* infection	<10^−6^	45.6 (0–84)	(0.09–53.34)	0.960	2/0.5946	0.0418	Yes	No/Yes	Ⅳ
	<10^−6^	47.4 (9–70)	(1.18, 4.06)	0.337	9/5.0139	0.0317	Yes	No/Yes	Ⅱ
Coinfection of HPV and *Chlamydia trachomatis*	<10^−6^	44.0 (0–78)	(1.31–14.66)	0.352	4/1.2393	0.0054	Yes	No/Yes	Ⅱ
*Chlamydia trachomatis* infection	<10^−6^	31.9 (0–67)	(1.48–2.95)	0.274	5/3.0626	0.1838	Yes	No/No	Ⅰ
	<0.05 but >0.001	0 (0–85)	(0.84–3.04)	0.122	2/0.5205	0.0279	No	No/Yes	Ⅳ
*Chlamydia trachomatis* infection (serum)	<10^−6^	39.6 (0–66)	(1.42–3.26)	0.481	9/4.5148	0.0116	Yes	No/Yes	Ⅱ
Epstein-Barr virus	<0.001 but >10^−6^	76.3 (60–86)	(0.25–63.55)	0.075	7/11.9431	N/A	Yes	Yes/No	Ⅳ
Cervicovaginal lactobacilli	<0.05 but >0.001	0 (0–90)	(0.00–343.66)	0.242	2/0.2197	0.0001	Yes	No/Yes	Ⅳ
**Reproductive factors**									
In vitro fertilization	>0.05	69 (10–89)	(0.03–34.71)	0.291	1/1.1316	0.8839	Yes	No/No	NS
Intrauterine device use	<0.001 but >10^−6^	42.5 (0–68)	(0.38–1.07)	0.029	5/4.9806	0.9902	No	Yes/No	Ⅲ
Oral contraceptives use	<0.05 but >0.001	67.7 (6–89)	(0.38–7.58)	0.232	3/1.1125	0.0352	Yes	No/Yes	Ⅳ
	<0.001 but >10^−6^	0 (0–85)	(1.06–2.96)	0.264	3/0.491	0.0001	Yes	No/Yes	Ⅳ
	<10^−6^	0 (0–79)	(1.12–1.50)	0.181	1/0.434	0.3686	Yes	No/No	Ⅰ
Oral contraceptive >10 years	<0.001 but >10^−6^	0 (0–90)	(0.13–38.57)	0.832	2/0.5229	0.0246	Yes	No/Yes	Ⅲ
**Diseases**									
Endometriosis	<0.001 but >10^−6^	0 (0–90)	(0.16–2.84)	0.713	2/0.2627	0.0004	Yes	No/Yes	Ⅲ
Gestational diabetes mellitus	>0.05	0 (0–90)	(0.23–4.49)	0.037	0/0.4407	0.4723	No	Yes/No	NS
**Nutrients and their levels**									
Total vitamin A intake	<10^−6^	77.9 (67–85)	(0.25–1.39)	0.380	9/11.0299	N/A	No	No/No	Ⅲ
Blood vitamin A levels (retinol)	>0.05	0.0 (0–90)	(0.15–8.93)	0.901	0/0.1579	0.6831	No	No/No	NS
Blood vitamin A levels (carotene)	<0.05 but >0.001	69.8 (23–88)	(0.10–2.29)	0.202	3/2.0479	0.3409	No	No/No	Ⅳ
Retinol intake	<0.05 but >0.001	41.3 (0–74)	(0.46–1.39)	0.054	2/1.8919	0.9283	No	Yes/No	Ⅳ
Carotene intake	<0.001 but >10^−6^	82.6 (67–91)	(0.15–1.75)	0.620	5/2.9124	0.1094	Yes	No/No	Ⅲ
Carotenoid intake	<0.05 but >0.001	52.5 (0–86)	(0.02–19.91)	0.931	2/0.6592	0.0615	Yes	No/Yes	Ⅳ
Retinol intake	>0.05	0 (0–90)	(0.13–5.32)	0.553	0/0.1833	0.6586	No	No/No	NS
Carotene intake	<0.05 but >0.001	78.8 (32–93)	(0.00–1239.10)	0.140	2/1.5758	0.6238	Yes	No/No	Ⅳ
Serum Selenium levels	<0.001 but >10^−6^	0 (0–79)	(0.35–0.86)	0.211	3/0.5576	0.0005	Yes	No/Yes	Ⅳ
Vitamin E	<0.001 but >10^−6^	77.6 (58–88)	(0.19–1.50)	0.767	5/3.8431	0.4130	Yes	No/No	Ⅲ

Abbreviation: CI, confidence interval; HPV, human papillomavirus.

^a^ *p* value under the random-effects model.

^b^ Expected number of statistically significant studies using the point estimate of the largest study (smallest standard error) as the plausible effect size.

^c^ Observed/Expected number of statistically significant studies.

^d^ *p* value of the excess statistical significance test.

^e^ Criteria for classification of the credibility of the evidence. Ⅰ, Strong; Ⅱ, Highly-suggestive evidence; Ⅲ, Suggestive evidence; Ⅳ, Weak evidence; NS, Non-significant associations.

All statistical tests two sided.

### Small-Study Effects and Excess Significance Bias

Out of the 39 meta-analyses, five (13%) associations met the criteria for small-study effects (Egger’s test *p* < 0.1, and effect size of the largest study smaller than the summary effect size); these included HPV and HPV16, Epstein-Barr virus, intrauterine device, gestational diabetes mellitus, and retinol intake. The 12 (30%) risk factors that had evidence of excess significance bias (based on O > E and *p* < 0.1) were: smoking, HPV and HPV16, *C. trachomatis*, cervicovaginal lactobacilli, oral contraceptives, endometriosis, carotenoid intake, and selenium intake (Table 3).

### Evaluation of Meta-Analysis Quality

The quality of the included meta-analyses was assessed on the basis of the AMSTAR tool (Supplementary Table S2). Overall, eight (44%) meta-analyses were scored as high quality (≥8 points), six (33%) were scored as moderate quality (4–7 points), and only four meta-analyses were scored as low quality (<4 points); quality scores for the included meta-analyses ranged from 2 to 9. Low quality meta-analyses investigated the following exposures: smoking, HPV and HPV16, and Epstein-Barr virus.

### Evaluation of Meta-Analysis Evidence

We used specific criteria to grade the 39 associations in the 18 meta-analyses. After applying our credibility criteria, only two associations (*C. trachomatis* infection and oral contraceptive use) presented strong evidence. Four associations presented highly suggestive evidence and assessed the association between extragenetic factors and CC risk, such as *C. trachomatis* (exposed vs. unexposed) and smoking (smokers vs. non-smokers). Six extragenetic factors (intrauterine devices, oral contraceptives, endometriosis, vitamin A, carotene, and vitamin E intake) presented suggestive evidence. In addition, 17 associations were supported by weak evidence and 10 associations presented no association. The detailed results of the analyses on which the evidence ratings were based are shown in Tables 2, 3.

## Discussion

### Main Findings

This is the first umbrella review to provide a comprehensive overview and a critical evaluation of the association between extragenetic factors and CC risk. In this umbrella review, by summarizing the evidence from related systematic reviews and meta‐analyses, we examined 39 different meta-analyses of different non-genetic risk factors and CC risk. Of these, two risk factors were supported by strong evidence (*C. trachomatis* infection and oral contraceptives). With the evaluation of substantial heterogeneity between studies, small study effects, and excess significance bias, we found that *C. trachomatis* infection and oral contraceptive use can increase CC risk. Furthermore, four associations (*C. trachomatis* and smoke) were supported by highly suggestive evidence, six associations (intrauterine devices, oral contraceptives, endometriosis, vitamin A, carotene, and vitamin E intake) were supported by suggestive evidence, and 17 associations were supported by weak evidence.

Our umbrella review of the existing evidence reports a positive association between *C. trachomatis* infection and CC risk. There was no evidence of small-study effects or excess significance bias for this association. Our results are consistent with several previous studies (50, 51). A large prospective cohort study based on the European Prospective Investigation into Cancer and Nutrition showed that previous exposure to *C. trachomatis* was strongly associated with invasive CC (50). Additionally, a cohort study of 8,812 women examined whether *C. trachomatis* was a potential cofactor in the development of cervical intraepithelial neoplasia grade 2 or higher, and found that *C. trachomatis* infection may facilitate the development of early cervical lesions (51). The mechanism of *C. trachomatis*-associated CC remains unclear. Yang et al. have shown that women infected with *C. trachomatis* have increased CC risk resulting from alterations in the apoptosis pathway, the DNA repair system, the protein folding response, and host intracellular protein targeting (52). In our review, studies with different classes of evidence for the same risk factor may be explained by inconsistencies in outcome. A stronger association may be observed between *C. trachomatis* infection and squamous carcinoma of the cervix than adenocarcinoma of cervix.

Our study found strong evidence for the association between oral contraceptive use and increased CC risk. This finding was consistent with a cohort study that was part of the UK Royal College of General Practitioners’ Oral Contraception Study, which found an increased risk of breast cancer and CC in current and recent oral contraceptive users (53). A pooled study from 24 studies worldwide revealed that the risk of invasive CC increased among current users of oral contraceptives with longer duration of use (54). A possible mechanism for the associations between oral contraceptive use and CC is that estrogens and progestogens may interact with hormone receptors, which are expressed in cervical tissue, and thereby affect the natural process of HPV infection (55). Women who had taken oral contraceptives for 5–9 years were nearly three times more likely than non-users to develop CC. The World Health Organization reported that CC risk did not change with respect to time since first or last oral contraceptive use, or with respect to age at first use (56). Compared with other articles, we met the criteria for strong evidence, which means the number of cases in our study was greater than 1,000, there was an absence of small-study effects, and no excess significance bias. In the future, more prospective cohort studies are needed to further evaluate this topic.

There was highly suggestive evidence that smoking was associated with CC risk. However, the association between smoking and CC may also carry excess significance bias, and should be interpreted carefully. Previous articles on smoking and CC risk are very common and cover all forms of smoking, such as active smoking, passive smoking, and exposure through semen of sexual partners who smoke. The study found that intraepithelial lesions were reported to have high frequency of malignancy in the study groups that were associated with active or passive tobacco use (57). Tobacco smoke is a cofactor; it can affect a plethora of signaling pathways involved in cancer initiation, promotion, and progression, and women who smoke are more susceptible to CC (58).

Our study also revealed that suggestive evidence supported the finding intrauterine device use and endometriosis are protective factors for CC. A cohort study with 1867 women reported that intrauterine device use was protective against CC (59). Data from a pooled analysis demonstrated that intrauterine device use may serve as a protective cofactor in the occurrence and development of CC (60). In addition, there was a reduced risk for CC (standardized incidence ratios 0.71) among women with endometriosis in a cohort study in Sweden (61). Owing to frequent gynecological examinations for endometriosis patients, endometriosis may indeed serve as a protective factor for the early diagnosis of CC (62).

Several studies were conducted to assess the association between nutrient intake and CC risk, though contradictory results have been reported and no certain conclusions have been reached until now. Our study found that intake of vitamin A, carotene, and vitamin E can reduce CC risk. For vitamin A, a case-control study in Korea reported a strong inverse relationship between total intake of vitamin A and CC risk (63). A more recent study provided evidence that high carotenoid intake (α-carotene, β-carotene, and lutein/zeaxanthin) reduces CC risk, especially for individuals exposed to passive smoking (64). For vitamin E, a meta-analysis that included case-control studies reported significant preventive effects on cervical neoplasm (cervical dysplasia, cervical carcinoma *in situ*, and invasive cancer) in the highest intake (or serum level) group of vitamin E (65). Vitamin E is a potent antioxidant with anti-neoplastic actions in the cervix; it acts by preventing reactive oxygen species from oxidizing cellular proteins and DNA.

This umbrella review is the first to systematically summarize the current evidence for the association between extragenetic factors and CC risk. First, we used standard approaches, such as a systematic search strategy of three literature databases followed by independent study extraction by two investigators. Next, we included information about all extragenetic factors and CC that we were of interest in each study, such as subgroup analysis and dose-response analysis. We then analyzed excess of statistical significance and small-study effects. Quality of methods (AMSTAR) was assessed by standard approaches. Of note, most of the meta-analyses we included were recognized as moderate-to-high quality.

Several possible limitations of this umbrella review should be considered. First, all of the studies we included were systematic reviews and meta-analyses. The reliability of the included meta-analyses is indirectly dependent upon the original studies; there was no feasible way for us to control bias from the original research. However, we use AMSTAR tool to ensure the quality of included studies. Second, some of the included meta-analyses had scores indicating low quality methodology. Few of the included meta-analyses considered grey literature and also did not present a list of excluded studies. Even so most articles are rated as moderate to high quality, which aggrandize the credibility to our results. Third, we calculated the summary effect size from a combination of studies with different measures, such as OR, RR, and HR. When the outcome is uncommon, OR is statistically similar to RR (66). Lastly, we included the most recently published meta-analyses. Our conclusions are drawn is based on 18 studies. Further studies are needed to better elucidate the association between extragenetic factors and CC risk in the future.

In conclusion, two risk factors (oral contraceptive use and *Chlamydia trachomatis* infection) were supported by strong evidence. Exposure to oral contraceptives or infection with *C. trachomatis* can increase CC risk. From studies with highly suggestive evidence and suggestive evidence, smoking is shown to be a risk factor for CC, whereas intrauterine device use, endometriosis, vitamin A, carotene, and vitamin E are protective factors.
