# Supplementary material for: Non-Genetic Factors and Risk of Cervical Cancer: An Umbrella Review of Systematic Reviews and Meta-Analyses of Observational Studies
Source: Int J Public Health. 2023 Mar 31;68:1605198. doi: 10.3389/ijph.2023.1605198 (PMC10103589; doi:10.3389/ijph.2023.1605198)
Supplement: Supplementary file 1 [file Table1.pdf]

**International Journal of Public Health**

**Review**

**Supplementary materials**

**Table S1. Search strategy used in the umbrella review (Liaoning, China. 2020)**

| |
|---|
| Literature search strategy in PubMed |
| ((cervical [title/abstract]) OR (uterine cervical [title/abstract])) AND ((cancer [title/abstract]) OR (tumor [title/abstract]) or (tumour [title/abstract]) or (neoplasm [title/abstract]) or (carcinoma [title/abstract])) and ((systematic review [title/abstract]) or (meta-analysis [title/abstract])) |
| Literature search strategy in Web of Science |
| 1: Search TS = (cervical OR uterine cervical) |
| 2: Search TS = (cancer OR tumor OR tumour OR neoplasm OR carcinoma) |
| 3: Search TS = (systematic review OR meta-analysis) |
| 4: #3 AND #2 AND #1 |
| Literature search strategy in EMBASE |
| 1: cervical:ab,ti |
| 2: “uterine cervical”:ab,ti |
| 3: #1 OR #2 |

| |
|---|
| 4: cancer:ab,ti |
| 5: tumor:ab,ti |
| 6: tumour:ab,ti |
| 7: neoplasm:ab,ti |
| 8: carcinoma:ab,ti |
| 9: #4 OR #5 OR #6 OR #7 OR #8 |
| 10: “systematic review”:ab,ti |
| 11: “meta analysis”:ab,ti |
| 12: #10 OR #11 |
| 13: #3 AND #9 AND #12 |

**Table S2. Detailed evaluation of the methodological quality with AMSTAR (Liaoning, China. 2021)**

| **Author** | AMSTAR | | | | | | | | | | | total | Quality |
|---|---|---|---|---|---|---|---|---|---|---|---|---|---|
| | 1-item | 2-item | 3-item | 4-item | 5-item | 6-item | 7-item | 8-item | 9-item | 10-item | 11-item | | |
| Asthana et al (10) | Yes | CA | Yes | No | No | Yes | Yes | Yes | Yes | Yes | Yes | 8 | high quality |
| Berrington de González et al (33) | CA | CA | No | CA | No | Yes | No | Yes | Yes | No | No | 3 | low quality |
| Cao et al (38) | CA | CA | Yes | CA | No | Yes | No | Yes | Yes | Yes | Yes | 6 | moderate quality |
| Cortessis et al (44) | No | CA | Yes | No | Yes | Yes | No | Yes | Yes | Yes | Yes | 7 | moderate quality |
| De Lima et al (41) | CA | CA | No | No | No | Yes | No | No | Yes | No | No | 2 | low quality |
| Hang et al (39) | CA | CA | No | No | No | Yes | No | No | Yes | Yes | Yes | 4 | moderate quality |
| He et al (48) | CA | Yes | Yes | CA | No | Yes | Yes | Yes | Yes | Yes | Yes | 8 | high quality |
| Hu et al (49) | Yes | Yes | Yes | No | No | Yes | Yes | Yes | Yes | Yes | Yes | 9 | high quality |
| Lehtinen et al (37) | CA | CA | No | CA | No | Yes | No | No | Yes | No | No | 2 | low quality |
| Li et al (45) | CA | CA | CA | CA | No | Yes | Yes | Yes | Yes | Yes | Yes | 6 | moderate quality |
| Li et al (43) | CA | Yes | Yes | CA | No | Yes | Yes | Yes | Yes | Yes | Yes | 8 | high quality |
| Poorolajal et al (35) | CA | Yes | Yes | CA | No | Yes | Yes | Yes | Yes | Yes | Yes | 8 | high quality |
| Appleby et al (34) | CA | CA | CA | CA | No | Yes | No | No | Yes | No | Yes | 3 | low quality |
| Sugawara et al (36) | CA | CA | Yes | No | No | Yes | No | Yes | Yes | Yes | Yes | 6 | moderate quality |
| Wang et al (42) | Yes | Yes | Yes | No | No | Yes | Yes | Yes | Yes | Yes | Yes | 9 | high quality |
| Wang et al (46) | CA | Yes | Yes | CA | No | Yes | Yes | Yes | Yes | Yes | Yes | 8 | high quality |
| Zhang et al (47) | CA | CA | Yes | No | No | Yes | Yes | Yes | Yes | Yes | Yes | 7 | moderate quality |
| Zhu et al (40) | Yes | CA | Yes | No | No | Yes | Yes | Yes | Yes | Yes | Yes | 8 | high quality |

**Table S3. The criteria for classification of the credibility of the evidence (Liaoning, China. 2022)**

| **Evidence** | **Criteria** |
| --- | --- |
| Strong | Summary random effect p value < $10^{-6}$ |
| | Cases > 1000 or participants > 20000 |
| | The largest study reported a significant effect ($p < 0.05$) |
| | 95% prediction interval excludes null value |
| | $I^2 < 50\%$ |
| | No evidence of small-study effects ($p > 0.1$) |
| | No evidence of excess of significance ($p > 0.1$) |
| Highly-suggestive evidence | Summary random effect p value < $10^{-6}$ |
| | Cases > 1000 or participants > 20000 |
| | The largest study reported a significant effect ($p < 0.05$) |
| Suggestive evidence | Summary random effect p value < $10^{-3}$ |
| | Cases > 1000 or participants > 20000 |
| Weak evidence | Summary random effect p value < 0.05 |
| Non-significant associations | Summary random effect p value > 0.05 |
